# Mixed Connective Tissue Disorder Complicated by Polymyositis, Sjogren’s Syndrome, Pleural Effusion and Pericarditis

**DOI:** 10.7759/cureus.906

**Published:** 2016-12-01

**Authors:** Maryam Kundi, Salman Assad, Sumbal Babar, Usman Ghani, Sahla Hammad, Ahmed G Sheikh, Asif K Kundi, Amjad Sheikh

**Affiliations:** 1 Department of Internal Medicine, Carthage Area Hospital, New York, USA; 2 Department of Medicine, Shifa Tameer-e-Millat University, Islamabad, Pakistan; 3 Khyber Medical University; 4 Department of Medicine, Shifa International Hospital, Islamabad, Pakistan; 5 Department of Internal Medicine, Shifa International Hospital, Islamabad, Pakistan; 6 Family Medicine Residency, UHS Wilson Medical Center Johnson City, NY

**Keywords:** connective tissue disease, steroids, pleural efffusion

## Abstract

We report a case of a 24-year-old female with a history of asthma and gastroesophageal reflux disease (GERD). She presented to the emergency room with severe chest pain, chest tightness, and shortness of breath following an upper respiratory tract infection. The patient reported that she had a cough and runny nose one week prior to this presentation, followed by a sudden sharp pain in the center of the chest 8/10 in intensity on the visual analog scale and pleuritic in nature, which aggravated by deep breathing and lying down flat. It was relieved by sitting up straight and did not radiate to her left arm or jaw. Computed tomography (CT) scan of the chest, posteroanterior and lateral views, showed a mild left pleural effusion with adjacent left basilar atelectasis/infiltrate. CT angiography of the chest with axial contrast showed mild left pleural effusion as well as a small pericardial effusion with bilateral lower lobe interstitial infiltrates. There was no evidence of pulmonary embolism. Electrocardiogram (EKG) showed no apparent ST segment elevation or depression that would be consistent with pericarditis, or acute ischemia or infarct. There was non-specific T wave abnormality. The patient was prescribed prednisone on a tapering dose. On follow-up visit, her condition significantly improved.

## Introduction

Mixed connective tissue disease (MCTD) was initially described as a unique syndrome with features of systemic lupus erythematosus (SLE), systemic sclerosis, and myositis associated with antibodies to a nuclear ribonucleoprotein, U1 ribonucleoprotein (RNP). Subsequently, the serological specificity was defined to epitopes on the 70 kD phosphoprotein uniquely associated with the ribonucleoprotein particle containing U1 RNP [1]. The original claims for MCTD have subsequently become points of contention. These include the clinical distinctiveness based on the presence of a particular group of features, notably Raynaud's phenomenon, polyarthritis, "puffy" hands, oesophageal dysmotility, myositis and lymphadenopathy in the absence of cerebral and renal disease or vasculitis, a benign prognosis, responsiveness to corticosteroids, and the presence of high titres of anti-U1 RNP [2].

## Case presentation

A 24-year-old female with a history of asthma and GERD presented to the emergency room with severe chest pain, chest tightness and shortness of breath following an upper respiratory tract infection. The patient reported episodes of dry cough and runny nose one week prior to this presentation, followed by a sudden sharp pain in the center of the chest. The pain was 8/10 in intensity on the visual analog scale and pleuritic in nature, which aggravated by deep breathing and lying down flat. It was relieved by sitting up straight and did not radiate to her left arm or jaw. The patient has associated cough and high-grade fever but denied chills, wheezing, orthopnea, nausea or vomiting. She had not experienced similar complaints in the past. The patient also complained of muscle aches along the back of her neck and shoulders. She denied smoking or alcohol use.

Physical examination revealed a healthy looking African American female. Auscultation of the chest and back demonstrated slightly diminished breath sounds at the bilateral lung bases. No murmurs or friction rubs were appreciated on precordial examination. Vital signs were recorded, monitored and charted. Initially, the heart rate was 105/min but later decreased to 94/min, temperature upon arrival to the emergency department was 103.3 °F and after treatment, it improved to 98.4 °F, oxygen (O_^2^_) saturation was 99% on room air and respiratory rate was 18/min.

Laboratory investigations showed low serum sodium levels suggesting hyponatremia secondary to hypovolemic state, high aspartate aminotransferase (AST) and alanine transaminase (ALT) levels, possibly due to an underlying infection (Table 1). The patient was anemic but white blood cell count was elevated at 10.6 k/mm^3^. Acute-phase reactants such as erythrocyte sedimentation rate, C-reactive protein, and ferritin levels were high. Creatine kinase (CK) was markedly elevated with a low CK-MB relative index indicative of skeletal muscle injury. Mild increase in troponin levels was suggestive of pericardial inflammation. Positive antinuclear antibodies (ANA) and elevated levels of anti-dsDNA, anti-RNP, anti-Smith and anti-SSA/B antibodies consistent with a mixed picture of systemic lupus erythematosus (SLE), Sjögren's syndrome and polymyositis were found. 

**Table 1:** Laboratory workup

Laboratory Variables	Values
Antinuclear antibodies (ANA)	Positive
Anti-Double stranded DNA antibodies (Anti-dsDNA)	12 IU/L
Anti-RNP antibodies	>8.0 AI
Anti-Smith antibodies	>8.0 AI
Anti-Sjögren's-syndrome antibodies (Anti-SS)	>0.2 AI
Serum sodium	126.1 mEq/L
Serum potassium	3.8 mEq/L
Serum calcium	8.4 mEq/L
AST/SGOT	185 U/L
ALT/SGPT	86 U/L
Direct bilirubin	0.3 mg/dL
Albumin	2.7 gm/dL
Creatine kinase (CK)	3744 U/L
CK-MB	3.7 ng/mL
CK-MB relative index	0.09
Creatinine	1.12 mg/dL
Myoglobin	>500 ng/mL
C- reactive protein	13.20 mg/dL
Erythrocyte sedimentation rate (ESR)	86 mm/hr
White blood cell count (WBC)	10.6 K/mm^3^
Neutrophils	91.28 %
Hemoglobin	11.7 g/dL
Mean corpuscular hemoglobin (MCH)	26.0 pg
Serum ferritin	115.3 ng/mL
Serum iron	18 ug/dL

**Figure 1:**
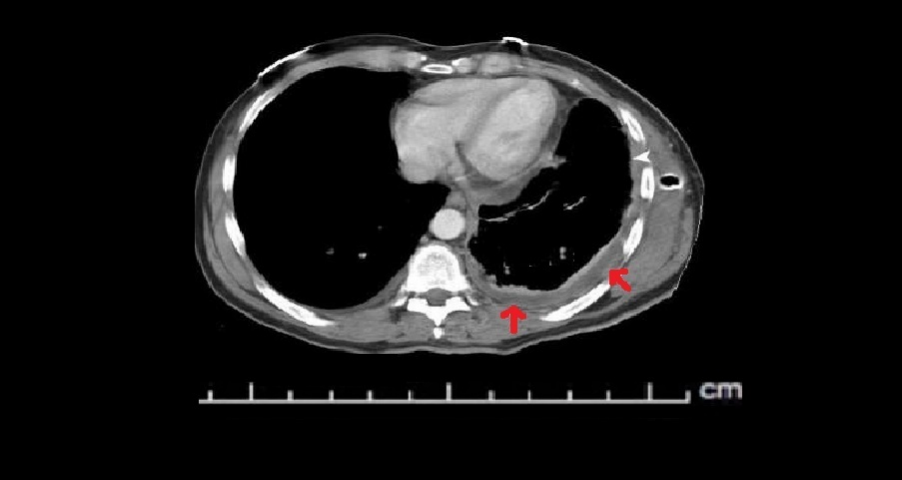
A computed tomography angiogram (CTA) showing mild left pleural effusion (red arrows) and a small pericardial effusion

**Figure 2:**
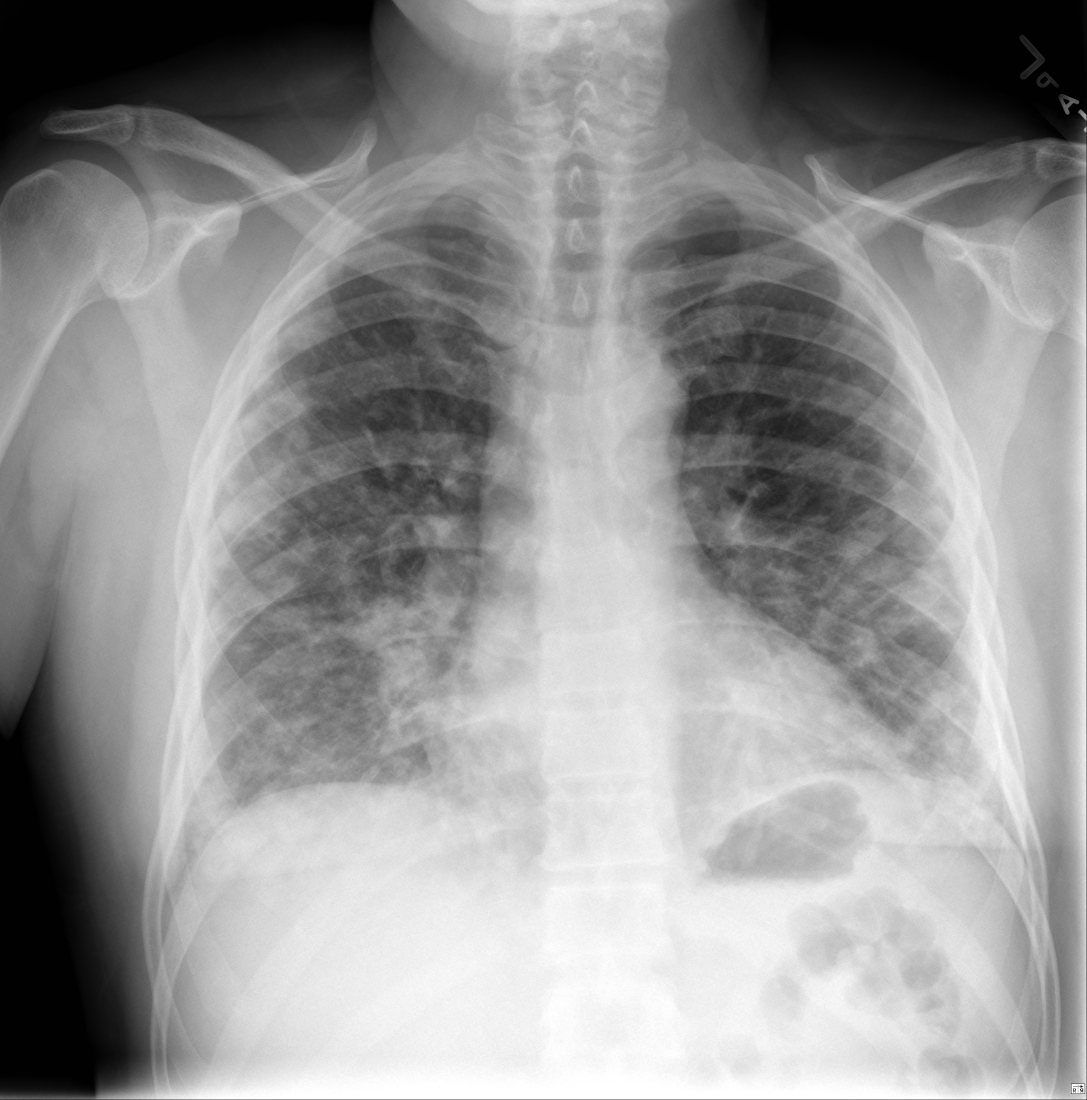
Chest X-ray (PA view): adjacent left basilar infiltrates and mild atelectasis. No infiltrates are seen on the right side

**Figure 3:**
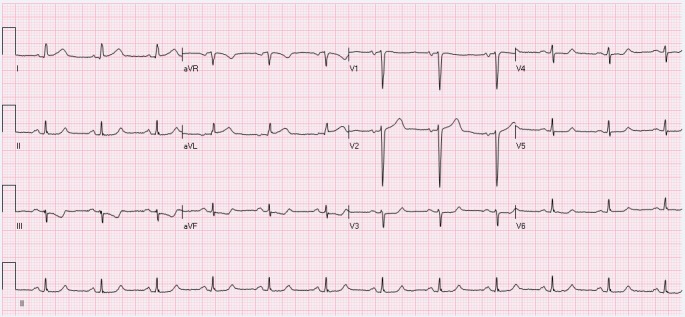
EKG showing non-specific T wave abnormality

The CT scan of chest and chest X-ray are shown in Figures 1-2. CT angiography of the chest was also performed. Axial contrast enhanced images using 100 Ml Isovue 370 intravenous contrast material with multiplanar reformations showed mild left pleural effusion as well as a small pericardial effusion with bilateral lower lobe interstitial infiltrates. There was no evidence of pulmonary embolism. Electrocardiogram (ECG) showed no apparent ST segment elevation or depression (Figure 3). Echocardiogram showed small pericardial effusion, no diastolic chamber collapse, ejection fraction of 65-70%, normal left ventricular diastolic function, normal left ventricular internal dimensions and wall thickness.

In the emergency department, she was diagnosed with pericarditis based on history, but imaging of the chest showed diffuse infiltrates in the lungs indicating multifocal community acquired pneumonia for which she was treated with 250 mg Zithromax (azithromycin) PO daily, 1 g Rocephin (ceftriaxone) IV twice a day and 2 litres of intravenous (IV) fluid. The patient received Dilaudid (hydromorphone) and Toradol (non-steroidal anti-inflammatory drug) for her chest pain and possible pericarditis. She was also managed with scheduled ibuprofen 600 mg every eight hours for generalized body aches, colchicine for polyserositis, and pantoprazole sodium sesquihydrate (proton pump inhibitor) 20 mg PO daily for GERD. She responded well to the medications. The patient was prescribed prednisone on a tapering dose. On the follow-up visit, her condition significantly improved although her creatinine phosphokinase levels remained elevated suggesting rhabdomyolysis and polymyositis. Informed consent was obtained from the patient for this study.

## Discussion

The universal definition of mixed connective tissue disease (MCTD) and its classification criteria are not established. Some experts describe MCTD as a syndrome with overlapping features of various connective tissue diseases (CTDs). Multiple case series and cohorts have described MCTD as a complex disease with variable clinical presentation [3-4]. The peak incidence of the disease occurs in the third decade of life and may affect people in all age groups. The annual incidence of MCTD in the United States is reported to be 1.9 per million, with a female predominance [5]. The diagnosis of MCTD can be challenging because of overlapping symptoms of systemic lupus erythematosus (SLE), systemic sclerosis (SSc) and polymyositis/dermatomyositis (PM/DM). The most common clinical features of MCTD reported are Raynaud’s phenomenon and 'puffy hands'. The cumulative frequency of these features is more than 90% in MCTD. Various diagnostic criteria have been established to aid in the diagnosis of MCTD. Diagnosis of MCTD should be considered in the presence of anti-RNP antibodies along with Raynaud’s phenomenon and swollen hands, along with the presence of (two or more) arthritis, pericarditis, myositis, interstitial lung disease (ILD) or esophageal dysmotility.

In this case report, the diagnostic challenge of variable symptom presentation and overlapping immunological workup suggested the involvement of multiple CTDs. The commonly used coexisting clinical criteria for the diagnosis of MCTD are the Alarcon-Segovia criteria and the Kasukawa criteria [6-7]. A major criterion included in the diagnostic set is myositis, with prevalence ranging 35-79% in patients with MCTD. It is rarely a presenting symptom and develops slowly over the course of the disease with a similar distribution pattern as in PM/DM associated muscle injury [8]. Joint pain (polyarthritis) is another major symptom listed in the criteria set and is commonly associated with MCTD. Lung manifestations in MCTD include interstitial lung disease (ILD) and pulmonary hypertension (PAH), with nonspecific interstitial pneumonia (NISP) being the most commonly observed histological pattern of ILD [9]. NISP presents as ground glass opacities and reticular pattern on high-resolution computed tomography (HRCT). Patients with lung involvement have lower pulmonary function test values. Gastrointestinal (GI) involvement includes esophageal dysmotility and hypomotility, which are also part of Kasukawa criteria set only. Gastroesophageal reflux disease is common and may increase the risk of aspiration syndromes. Pleural thickening or effusion occurs in less than 10% of patients.

Skin involvement is included in the Kasukawa criteria set. Studies report a cumulative frequency of 36-53% of skin manifestations over the course of the disease, including photosensitivity, malar rash, telangiectasia, or hypo- and hyper-pigmentation. Also, 20% of patients with MCTD develop renal abnormalities, most commonly immune complex depositions (membranous glomerular nephropathy); however, this is not a part of the diagnostic criteria sets. In this case report, the diagnostic features were variable and did not meet a particular criteria set. Our patient had a combination of gastroesophageal reflux disease, pericarditis, serositis (pleural effusion) and myositis, which along with the positive immunological workup was suggestive of MCTD. The autoimmune workup was positive for ANA, anti-dsDNA, anti-Sm and anti-SSA/B; however, the presence of anti-RNP was most suggestive of MCTD. 

## Conclusions

When overlapping features of the autoimmune disease are present, MCTD should be considered as a possible differential diagnosis. Prognosis of such patients depends on earlier diagnosis and treatment with steroids to prevent further progression.
